# Isoflavone and Soyfood Intake and Colorectal Cancer Risk: A Case-Control Study in Korea

**DOI:** 10.1371/journal.pone.0143228

**Published:** 2015-11-17

**Authors:** Aesun Shin, Jeonghee Lee, Jeeyoo Lee, Moon Sung Park, Ji Won Park, Sung Chan Park, Jae Hwan Oh, Jeongseon Kim

**Affiliations:** 1 Department of Preventive Medicine, Seoul National University College of Medicine, Seoul, 110–799, Republic of Korea; 2 Molecular Epidemiology Branch, National Cancer Center, Goyang-si, 410–769, Republic of Korea; 3 Department of Nutritional Science and Food Management, Ewha Womans University, Seoul, 120–750, Republic of Korea; 4 Gachon University College of Nursing, Incheon, 406–799, Republic of Korea; 5 Department of Surgery, Seoul National University College of Medicine and Hospital, Seoul, 110–799, Republic of Korea; 6 Center for Colorectal Cancer, National Cancer Center, Goyang-si, 410–769, Republic of Korea; Baylor University Medical Center, UNITED STATES

## Abstract

We aimed to assess the relationship between dietary soyfood and isoflavone intake and colorectal cancer risk in a case-control study. A total of 901 colorectal cancer cases and 2669 controls were recruited at the National Cancer Center, Korea. A semi-quantitative food frequency questionnaire was used to assess the usual dietary habits, and the isoflavone intake level was estimated from five soyfood items. A high intake of total soy products, legumes, and sprouts was associated with a reduced risk for colorectal cancer in men and women, although the middle quartiles of intake of total soy products were associated with an elevated risk. In contrast, a high intake of fermented soy paste was associated with an elevated risk for colorectal cancer in men. The groups with the highest intake quartiles of isoflavones showed a decreased risk for colorectal cancer compared to their counterparts with the lowest intake quartiles in men (odds ratio (OR): 0.67, 95% confidence interval (CI): 0.51–0.89) and women (OR: 0.65, 95% CI: 0.43–0.99). The reduced risk for the highest intake groups persisted for distal colon cancer in men and rectal cancer in women. The association between soyfood intake and colorectal cancer risk was more prominent among post-menopausal women than pre-menopausal women. In conclusion, a high intake of total soy products or dietary isoflavones was associated with a reduced risk for overall colorectal cancer, and the association may be more relevant to distal colon or rectal cancers.

## Introduction

Colorectal cancer is the third most common cancer in Korea, and the incidence rates have increased in recent decades.[1] Although the mortality rate of colorectal cancer in men has been stabile since 2002 and has decreased since 2004 in women,[2] colorectal cancer is the fourth most common cause of cancer death in Korea.[3] Risk factors such as body size, alcohol consumption, elevated fasting glucose and total cholesterol have defined colorectal cancer risk in Korea.[4, 5] Dietary factors may explain up to one-third of colorectal cancer risk,[6] however, epidemiological studies on the role of dietary habits on colorectal cancer risk in Korea are limited.[7–9]

Genistein, the major isoflavone in soy, has anti-carcinogenic effects in animal models,[10] and the protective effect of soyfood or isoflavone intake in the gastric and breast cancer risk has been reported in a Korean population.[11, 12] A meta-analysis of four cohort studies and seven case-control studies of soy and isoflavone intake and colorectal cancer risk concluded that there was no association between soy intake and colorectal cancer risk in men; however, the analysis suggested that there was a 21% reduction in the colorectal cancer risk of women when the highest *vs*. the lowest reported intake categories were compared.[13] However, substantial variation in the soy and isoflavone intake level among different populations ensures that the interpretation of the association is difficult.[14–18] In addition, increasing evidence suggests a differential risk factor profile for the subsites of colorectal cancer. [4, 19]

The aim of this study was to assess the association between dietary soy and isoflavone intake and colorectal cancer risk in a case-control study, taking into account the differences in that association between colorectal cancer subsites.

## Materials and Methods

### Study participants

Newly diagnosed colorectal cancer patients were contacted when they were admitted for treatment at the Center for Colorectal Cancer, National Cancer Center, Korea between August 2010 and August 2013. Among 1,427 eligible patients, 1,259 patients were contacted, and 1,070 patients agreed to participate in the study and provided informed consent. Among them, 925 completed a 106-food item semi-quantitative food frequency questionnaire (SQFFQ). The controls were selected from subjects who visited the same hospital for a health check-up provided by the National Health Insurance Cooperation, which covers the entire Korean population, during the identical time period. Up to 3 controls per case were matched by gender and 5 years of age groups. All participants provided written informed consent to participate, and the study protocol was approved by the institutional review board of the National Cancer Center (IRB No. NCCNCS-10-350).

### Dietary assessment

A trained dietitian conducted a face-to-face interview to collect information on lifestyle factors and dietary habits before a cancer diagnosis. Information on demographic and lifestyle risk factors were collected using a structured questionnaire. The dietary intake was assessed using a semi-quantitative food frequency questionnaire (SQFFQ) with a total of 106 food items. The validity and reproducibility of the questionnaire were reported previously.[20] The five soyfood items included in the SQFFQ were used for estimation of the isoflavone intake level using a Korean isoflavone database.[21] The five soyfood items were as follows: legumes (black soybeans and green peas), tofu (soybean curd), soy milk, sprouts (mung bean sprouts and soybean sprouts) and fermented soy paste (soybean paste and fermented soybeans).

### Statistical analysis

Three colorectal cancer patients with implausible energy intake (<500 kcal/day or >4000 kcal/day) were excluded from the analysis. The characteristics of the cases and controls were compared by Pearson’s chi-square statistics. The residual method was used to adjust the total energy intake.[22] The intake levels of soyfoods and isoflavones were categorized into quartiles according to the distribution of the control groups, except for soy milk, which showed a low proportion of consumption. The association between dietary factors and colorectal cancer risk was assessed by analysis of logistic regression models with adjustment for potential confounding variables, and odds ratios (OR) and their 95% confidence intervals (CI) were calculated. For the subsite analysis, polytomous logistic regression models were used. All analyses were performed separately by gender, and SAS version 9.3 software (SAS Institute, Inc., Cary, NC, USA) was used.

## Results

Although age was matched by a 10-year frequency, more male patients over 60 years of age were recruited. Male colorectal cancer patients were more likely to have less education and a lower household income. The patients were less like to participate in regular physical activity, and they had a high proportion of colorectal cancer in their family histories. Similar trends were observed for education, household income, and physical activity among the women. In addition, the female patients were more likely to have a body mass index of 25 kg/m^2^ or more or to ever have smoked or consumed alcohol (Table 1).

**Table 1:** General characteristics of colorectal cancer cases and controls; N (%).

	Male (n = 2,496)			Female (n = 1,192)		
	Case (n = 624)	Control (n = 1,872)	*P-value* ^1)^	Case (n = 298)	Control (n = 894)	*P-value* ^1)^
Age group (years)						
-49	128(20.5)	461(24.6)	< .001	82(27.5)	246(27.5)	1.000
50–59	226(36.2)	815(43.5)		111(37.3)	333(37.3)	
60+	270(43.3)	596(31.8)		105(35.2)	315(35.2)	
Marital status						
Married	556(89.1)	1,671(89.3)	0.262	216(72.5)	697(78.0)	0.028
Single	66(10.6)	167(8.9)		80(26.9)	184(20.6)	
Missing	2 (0.3)	34(1.8)		2(0.7)	13(1.5)	
Education level						
Under middle school	183(29.3)	250(13.4)	< .001	138(46.3)	146(16.3)	< .001
High school	265(42.5)	498(26.6)		103(34.6)	365(40.8)	
College or more	176(28.2)	1,024(54.7)		57(19.1)	332(37.1)	
Missing	0(0.0)	100(5.3)		0(0.0)	51(5.7)	
Household income (1,000won/month)						
<200	222(35.6)	348(18.6)	< .001	99(33.2)	212(23.7)	0.016
200–400	252(40.4)	727(38.8)		134(45.0)	313(35.0)	
>400	150(24.0)	588(31.4)		65(21.8)	230(25.7)	
Missing	0(0.0)	209(11.2)		0(0.0)	139(15.6)	
Body mass index (kg/m^2^)						
<25	431(69.1)	1,136(60.7)	< .001	207(69.5)	661(73.9)	0.133
≥25	192(30.8)	736(39.3)		91(30.5)	233(26.1)	
Missing	1(0.2)	0(0.0)		0(0.0)	0(0.0)	
Smoking status						
Non-smoker	145(23.2)	391(20.9)	0.418	264(88.6)	854(95.5)	< .001
Ex-smoker	302(48.4)	951(50.8)		15(5.0)	21(2.4)	
Current smoker	177(28.4)	530(28.3)		19(6.4)	18(2.0)	
Missing	0(0.0)	0(0.0)		0(0.0)	1(0.1)	
Alcohol consumption						
Non-drinker	107(17.2)	308(16.5)	< .001	172(57.7)	567(63.4)	0.031
Ex-drinker	103(16.5)	201(10.7)		26(8.7)	44(4.9)	
Current drinker	414(66.4)	1,360(72.7)		100(33.5)	282(31.5)	
Missing	0(0.0)	3(0.2)		0(0.0)	1(0.1)	
Regular exercise						
No	388(62.2)	786(42.0)	< .001	225(75.5)	403(45.1)	< .001
Yes	236(37.8)	1,073(57.3)		73(24.5)	488(54.6)	
Missing	0(0.0)	13(0.7)		0(0.0)	3(0.3)	
Family history of cancer						
No	391(62.7)	1,016(54.3)	< .001	171(57.4)	446(49.9)	0.038
Yes	233(37.3)	846(45.2)		127(42.6)	438(49.0)	
Missing	0(0.0)	10(0.5)		0(0.0)	10(1.1)	
Family history of colorectal cancer						
No	559(89.6)	1,768(94.4)	< .001	277(93.0)	816(91.3)	0.715
Yes	65(10.4)	94(5.0)		21(7.1)	68(7.6)	
Missing	0(0.0)	10(0.5)		0(0.0)	10(1.1)	

^1)^ p-values were calculated by chi-square test

The most abundant dietary source of isoflavones for men and women was soybean curd (tofu), which amounted to 38~44% of the isoflavones consumed, followed by black soybeans, soybean paste, and soybean milk. Daidzein and genistein were the most abundant isoflavones in men and women (data not shown). For the total soyfood, the group with the second and the third quartiles of intake showed increased risk for colorectal cancer in men, whereas a reduced risk for colorectal cancer among the highest intake groups was observed for both men (OR: 0.67, 95% CI: 0.49–0.92) and women (OR: 0.62, 95% CI: 0.39–1.0) (Table 2). Legumes and sprouts intake was inversely associated with colorectal cancer risk in both men and women, whereas fermented soy paste was associated with increased risk for colorectal cancer in men. Middle intake groups for tofu and soy milk showed increased risk for colorectal cancer in both men and women. Compared with the lowest intake quartiles group, the groups with the highest intake quartiles of total isoflavones showed a decreased risk for colorectal cancer in men (OR: 0.71, 95% CI: 0.52–0.97). A separate analysis of three isoflavones (daidzein, genistein, and glycitein) yielded results very similar to those for the total isoflavones.

**Table 2:** Odds ratios (OR) and 95% confidence intervals (Cl) of colorectal cancer risk in relation to intake of soy products and isoflavones.

	Men (n = 2,496)				Women (n = 1,192)		
	Controls/cases(n)	Age-adjusted OR(95%CI)	Multivariate OR^a^(95%CI)		Controls/cases(n)	Age-adjusted OR(95%CI)	Multivariate OR^a^(95%CI)
Total soy products (g/day)				Total soy products (g/day)			
Q1 (<40.34)	468/144	1.00	1.00	Q1 (<42.77)	224/72	1.00	1.00
Q2 (40.34-<64.64)	468/194	1.28(1.00–1.65)	1.38(1.05–1.80)	Q2 (42.77-<70.29)	223/96	1.34(0.93–1.91)	1.27(0.86–1.88)
Q3 (64.64-<105.03)	468/201	1.34(1.04–1.72)	1.40(1.07–1.83)	Q3 (70.29-<113.66)	223/91	1.27(0.88–1.82)	1.37(0.92–2.04)
Q4 (≥105.03)	468/85	0.56(0.41–0.75)	0.67(0.49–0.92)	Q4 (≥113.66)	224/39	0.54(0.35–0.83)	0.62(0.39–1.00)
P-value for trend		< .001	0.002	P-value for trend		0.001	0.028
Legumes (g/day)				Legumes (g/day)			
Q1 (<1.11)	468/242	1.00	1.00	Q1 (<1.22)	223/120	1.00	1.00
Q2 (1.11-<2.09)	468/131	0.52(0.41–0.67)	0.54(0.41–0.70)	Q2 (1.22-<2.40)	224/74	0.61(0.43–0.86)	0.68(0.46–0.99)
Q3 (2.09-<4.19)	468/123	0.48(0.37–0.61)	0.50(0.38–0.66)	Q3 (2.40-<5.82)	223/50	0.41(0.28–0.60)	0.45(0.30–0.69)
Q4 (≥4.19)	468/128	0.47(0.37–0.61)	0.49(0.37–0.64)	Q4 (≥5.82)	224/54	0.44(0.30–0.64)	0.47(0.31–0.71)
P-value for trend		< .001	0.001	P-value for trend		0.001	0.004
Tofu (g/day)				Tofu (g/day)			
Q1 (<17.19)	468/112	1.00	1.00	Q1 (<18.73)	224/59	1.00	1.00
Q2 (17.19-<30.77)	468/185	1.65(1.26–2.15)	1.82(1.37–2.42)	Q2 (18.73-<32.68)	223/95	1.62(1.11–2.35)	1.80(1.19–2.72)
Q3 (30.77-<52.86)	468/228	1.92(1.47–2.49)	2.03(1.54–2.69)	Q3 (32.68-<54.91)	223/92	1.57(1.08–2.28)	1.80(1.20–2.72)
Q4 (≥52.86)	468/99	0.87(0.64–1.17)	0.94(0.68–1.29)	Q4 (≥54.91)	223/92	0.88(0.58–1.34)	0.97(0.61–1.52)
P-value for trend		0.036	0.093	P-value for trend		0135	0.295
Soy milk (g/day)				Soy milk (g/day)			
None (= 0)	1068/360	1.00	1.00	None (= 0)	562/189	1.00	1.00
Low (0<-21.35)	402/207	1.59(1.29–1.95)	1.74(1.39–2.17)	Low (0<-19.1)	166/70	1.25(0.91–1.74)	1.50(1.04–2.15)
High (≥21.35)	402/57	0.41(0.31–0.56)	0.47(0.34–0.64)	High (≥19.1)	166/39	0.70(0.47–1.03)	0.82(0.54–1.25)
P-value for trend		< .001	< .001	P-value for trend		0.037	0.193
Sprouts (g/day)				Sprouts (g/day)			
Q1 (<5.29)	468/223	1.00	1.00	Q1 (<5.21)	223/92	1.00	1.00
Q2 (5.29-<10.67)	468/204	0.93(0.74–1.17)	0.99(0.77–1.26)	Q2 (5.21-<11.24)	224/94	1.02(0.72–1.43)	0.87(0.66–1.42)
Q3 (10.67-<20.50)	468/139	0.64(0.50–0.82)	0.69(0.53–0.91)	Q3 (11.24-<21.86)	223/76	0.83(0.58–1.18)	0.77(0.52–1.14)
Q4 (≥20.50)	468/58	0.26(0.18–0.35)	0.29(0.21–0.40)	Q4 (≥21.86)	224/36	0.39(0.25–0.60)	0.39(0.25–0.62)
P-value for trend		< .001	< .001	P-value for trend		< .001	< .001
Fermented soy paste (g/day)				Fermented soy paste (g/day)			
Q1 (<1.95)	468/93	1.00	1.00	Q1 (<2.08)	223/52	1.00	1.00
Q2 (1.95-<4.04)	468/159	1.70(1.27–2.26)	1.81(1.33–2.45)	Q2 (2.08-<4.50)	224/88	1.69(1.14–2.49)	1.62(1.06–2.49)
Q3 (4.04-<8.32)	468/186	1.91(1.44–2.53)	2.06(1.53–2.78)	Q3 (4.50-<8.70)	223/86	1.66(1.12–2.45)	1.57(1.02–2.41)
Q4 (≥8.32)	468/186	1.86(1.40–2.47)	1.82(1.35–2.46)	Q4 (≥8.70)	224/72	1.38(0.92–2.08)	1.22(0.77–1.91)
P-value for trend		0.002	0.012	P-value for trend		0.615	0.874
Isoflavone mg/day)				Isoflavone (mg/day)			
Q1 (<7.63)	468/142	1.00	1.00	Q1 (<8.08)	223/72	1.00	1.00
Q2 (7.63-<12.56)	468/186	1.26(0.97–1.62)	1.34(1.02–1.76)	Q2 (8.08<13.83)	224/103	1.42(1.00–2.02)	1.14(0.98–2.13)
Q3 (12.56-<20.89)	468/195	1.28(1.00–1.66)	1.37(1.04–1.79)	Q3 (13.83-<22.35)	224/76	1.04(0.72–1.52)	1.12(0.74–1.70)
Q4 (≥20.89)	468/101	0.65(0.48–0.86)	0.71(0.52–0.97)	Q4 (≥22.35)	223/47	0.65(0.43–0.98)	0.78(0.50–1.23)
P-value for trend^b^		< .001	0.005	P-value for trend		0.004	0.087
Daidzein (mg/day)				Daidzein (mg/day)			
Q1 (<3.20)	468/138	1.00	1.00	Q1 (<3.38)	223/74	1.00	1.00
Q2 (3.20-<5.28)	468/182	1.25(0.96–1.61)	1.28(0.97–1.69)	Q2 (3.38-<5.89)	224/104	1.40(0.98–1.98)	1.45(0.98–2.14)
Q3 (5.28-<9.04)	468/194	1.30(1.01–1.68)	1.39(1.06–1.82)	Q3 (5.89-<9.89)	224/68	0.91(0.62–1.33)	1.02(0.70–1.55)
Q4 (≥9.04)	468/110	0.71(0.54–0.95)	0.77(0.57–1.04)	Q4 (≥9.89)	223/52	0.70(0.46–1.04)	0.80(0.51–1.26)
P-value for trend		0.003	0.017	P-value for trend		0.007	0.076
Genistein (mg/day)				Genistein (mg/day)			
Q1 (<3.33)	468/136	1.00	1.00	Q1 (<3.56)	224/69	1.00	1.00
Q2 (3.33-<5.57)	468/168	1.18(0.91–1.53)	1.25(0.95–1.65)	Q2 (3.56-<6.08)	223/97	1.41(0.98–2.02)	1.47(0.99–2.18)
Q3 (5.57-<9.09)	468/209	1.43(1.11–1.84)	1.46(1.11–1.91)	Q3 (6.08-<9.79)	224/83	1.20(0.83–1.74)	1.20(0.80–1.81)
Q4 (≥9.09)	468/111	0.75(0.57–1.00)	0.84(0.62–1.14)	Q4 (≥9.79)	223/49	0.71(0.47–1.07)	0.86(0.55–1.35)
P-value for trend		0.028	0.155	P-value for trend		0.022	0.215
Glycitein (mg/day)				Glycitein (mg/day)			
Q1 (<0.85)	468/144	1.00	1.00	Q1 (<0.93)	224/84	1.00	1.00
Q2 (0.85-<1.38)	468/197	1.32(1.03–1.70)	1.45(1.11–1.90)	Q2 (0.93-<1.43)	223/78	0.93(0.65–1.34)	1.05(0.71–1.56)
Q3 (1.38-<2.21)	468/205	1.36(1.06–1.75)	1.49(1.14–1.94)	Q3 (1.43-<2.44)	223/103	1.23(0.87–1.73)	1.37(0.94–2.00)
Q4 (≥2.21)	468/78	0.52(0.38–0.71)	0.60(0.44–0.83)	Q4 (≥2.44)	224/33	0.39(0.25–0.61)	0.47(0.29–0.76)
P-value for trend		< .001	< .001	P-value for trend		< .001	0.004

^a^ Adjusted by age, education, alcohol consumption, and regular exercise

^b^ Test for trend calculated with the median intake for each category of soybean products or isoflavone intake as a continuous variable.

In the subsite analysis, a high consumption of total soy products and legumes was associated with the risk for distal colon cancer in men (Table 3) and rectal cancer in women (Table 4). However, similar to the results for overall colorectal cancer, the second and the third quartiles of intake groups for total soy products or tofu showed an elevated risk for proximal colon cancer, and rectal cancer in men and distal colon cancer in women. Soy milk was associated with a reduced risk of cancer in all the subsites in men, whereas no apparent association was observed for each subsite in women. Sprouts were associated with a reduced risk for all the subsites in men and women. The reduced risk for the highest intake groups of total isoflavones, daidzein, genistein, and glycitein persisted for distal colon cancer in men (Table 3) and rectal cancer in women (Table 4).

**Table 3:** Odds ratios (OR) and 95% confidence intervals (Cl) of colorectal cancer risk by subsites in relation to intake of soy products and isoflavones in men.

	Control (N = 1,872)	Proximal colon (N = 113)			Distal colon (N = 178)			Rectum (N = 320)		
	No	No	Age-adjusted OR(95%CI)	Multivariate OR^a^(95%CI)	No	Age-adjusted OR(95%CI)	Multivariate OR^a^(95%CI)	No	Age-adjusted OR(95%CI)	Multivariate OR^a^(95%CI)
Total soy products (g/day)										
Q1 (<40.34)	468	16	1.00	1.00	53	1.00	1.00	71	1.00	1.00
Q2 (40.34-<64.64)	468	40	2.37(1.31–4.30)	2.55(1.40–4.67)	47	0.84(0.55–1.27)	0.90(0.59–1.37)	100	1.35(0.97–1.88)	1.47(1.04–2.08)
Q3 (64.64-<105.03)	468	35	2.10(1.14–3.84)	2.22(1.20–4.10)	62	1.12(0.76–1.65)	1.18(0.79–1.76)	103	1.40(1.01–1.94)	1.46(1.04–2.07)
Q4 (≥105.03)	468	22	1.30(0.67–2.50)	1.54(0.79–3.00)	16	0.28(0.16–0.50)	0.33(0.18–0.58)	46	0.62(0.42–0.91)	0.76(0.50–1.14)
P-value for trend			0.660	0.906		< .001	< .001		0.002	0.049
Legumes (g/day)										
Q1 (<1.11)	468	42	1.00	1.00	73	1.00	1.00	122	1.00	1.00
Q2 (1.11-<2.09)	468	23	0.53(0.31–0.89)	0.53(0.31–0.90)	41	0.54(0.36–0.81)	0.56(0.37–0.84)	64	0.51(0.36–0.70)	0.52(0.37–0.73)
Q3 (2.09-<4.19)	468	24	0.53(0.31–0.89)	0.55(0.32–0.94)	35	0.45(0.29–0.68)	0.47(0.35–0.70)	60	0.46(0.33–0.65)	0.49(0.36–0.70)
Q4 (≥4.19)	468	24	0.50(0.30–0.85)	0.51(0.30–0.88)	29	0.35(0.22–0.55)	0.57(0.41–0.80)	74	0.55(0.40–0.75)	0.57(0.41–0.80)
P-value for trend			0.114	0.128		0.001	0.001		0.065	0.111
Tofu (g/day)										
Q1 (<17.19)	468	17	1.00	1.00	40	1.00	1.00	52	1.00	1.00
Q2 (17.19-<30.77)	468	31	1.81(0.99–3.32)	1.95(1.06–3.61)	50	1.24(0.80–1.92)	1.35(0.87–2.11)	99	1.89(1.32–2.71)	2.12(1.46–3.09)
Q3 (30.77-<52.86)	468	40	2.19(1.22–3.93)	2.29(1.26–4.14)	59	1.37(0.90–2.10)	1.45(0.94–2.24)	124	2.26(1.60–3.21)	2.42(1.68–3.49)
Q4 (≥52.86)	468	25	1.43(0.76–2.69)	1.54(0.81–2.92)	29	0.71(0.43–1.16)	0.76(0.46–1.26)	45	0.85(0.56–1.29)	0.92(0.60–1.42)
P-value for trend			0.619	0.501		0.086	0.130		0.006	0.121
Soy milk (g/day)										
None (= 0)	1068	64	1.00	1.00	117	1.00	1.00	2175	1.00	1.00
Low (0<-21.35)	402	35	1.52(0.99–2.33)	1.65(1.06–2.55)	50	1.18(0.83–1.68)	1.27(0.89–1.83)	113	1.78(1.36–2.32)	2.00(1.51–2.64)
High (≥21.35)	402	14	0.57(0.32–1.03)	0.63(0.35–1.15)	11	0.25(0.13–0.46)	0.27(0.14–0.50)	32	0.48(0.32–0.71)	0.55(0.37–0.83)
P-value for trend			0.023	0.055		< .001	< .001		< .001	< .001
Sprouts (g/day)										
Q1 (<5.29)	468	50	1.00	1.00	59	1.00	1.00	108	1.00	1.00
Q2 (5.29-<10.67)	468	22	0.45(0.27–0.75)	0.47(0.28–0.80)	71	1.22(0.85–1.77)	1.30(0.89–1.90)	109	1.02(0.76–1.38)	1.09(0.80–1.49)
Q3 (10.67-<20.50)	468	26	0.53(0.33–0.87)	0.58(0.35–0.95)	39	0.67(0.44–1.03)	0.74(0.48–1.14)	69	0.65(0.47–0.91)	0.72(0.51–1.01)
Q4 (≥20.50)	468	15	0.30(0.16–0.54)	0.33(0.18–0.59)	9	0.15(0.07–0.31)	0.17(0.08–0.35)	34	0.31(0.21–0.47)	0.36(0.24–0.54)
P-value for trend			< .001	0.001		< .001	< .001		< .001	< .001
Fermented soy paste (g/day)										
Q1 (<1.95)	468	14	1.00	1.00	29	1.00	1.00	47	1.00	1.00
Q2 (1.95-<4.04)	468	29	2.05(1.07–3.94)	2.13(1.10–4.11)	47	1.61(0.99–2.60)	1.71(1.04–2.79)	79	1.67(1.14–2.45)	1.78(1.20–2.66)
Q3 (4.04-<8.32)	468	32	2.17(1.14–4.13)	2.33(1.22–4.46)	49	1.61(0.99–2.59)	1.73(1.07–2.82)	100	2.04(1.41–2.96)	2.21(1.50–3.25)
Q4 (≥8.32)	468	38	2.50(1.33–4.71)	2.45(1.30–4.65)	53	1.69(1.05–2.72)	1.68(1.03–2.72)	94	1.87(1.29–2.73)	1.82(1.23–2.69)
P-value for trend			0.020	0.033		0.110	0.173		0.014	0.053
Isoflavone (mg/day)										
Q1 (<7.63)	468	19	1.00	1.00	53	1.00	1.00	66	1.00	1.00
Q2 (7.63-<12.56)	468	35	1.76(0.99–3.13)	1.90(1.06–3.39)	48	0.86(0.57–1.30)	0.93(0.61–1.41)	95	1.39(0.99–1.95)	1.50(1.05–2.15)
Q3 (12.56-<20.89)	468	34	1.67(0.93–2.97)	1.79(1.00–3.23)	58	1.01(0.68–1.51)	1.08(0.72–1.62)	103	1.47(1.05–2.06)	1.57(1.10–2.24)
Q4 (≥20.89)	468	25	1.19(0.64–2.20)	1.29(0.69–2.42)	19	0.32(0.19–0.55)	0.34(0.20–0.59)	56	0.78(0.53–1.14)	0.88(0.56–1.32)
P-value for trend^b^			0.869	0.940		< .001	< .001		0.058	0.215
Daidzein (mg/day)										
Q1 (<3.20)	468	20	1.00	1.00	49	1.00	1.00	68	1.00	1.00
Q2 (3.20-<5.28)	468	34	1.60(091–2.83)	1.65(0.93–2.94)	49	0.94(0.62–1.42)	0.97(0.63–1.48)	88	1.23(0.87–1.74)	1.28(0.89–1.83)
Q3 (5.28-<9.04)	468	32	1.48(0.83–2.63)	1.58(0.88–2.84)	58	1.08(0.72–1.62)	1.15(0.76–1.75)	104	1.43(1.02–2.00)	1.53(1.09–2.18)
Q4 (≥9.04)	468	27	1.20(0.66–2.19)	1.27(0.69–2.34)	22	0.40(0.23–0.67)	0.41(0.24–0.70)	60	0.80(0.55–1.16)	0.88(0.59–1.30)
P-value for trend			0.955	0.890		< .001	0.001		0.106	0.284
Genistein (mg/day)										
Q1 (<3.33)	468	18	1.00	1.00	50	1.00	1.00	64	1.00	1.00
Q2 (3.33-<5.57)	468	32	1.69(0.93–3.01)	1.81(0.99–3.30)	40	0.76(0.49–1.17)	0.80(0.51–1.26)	89	1.33(0.94–1.89)	1.44(1.00–2.07)
Q3 (5.57-<9.09)	468	35	1.80(1.003.23)	1.86(1.03–3.38)	61	1.12(0.75–1.67)	1.16(0.77–1.74)	112	1.64(1.17–2.29)	1.68(1.18–2.39)
Q4 (≥9.09)	468	28	1.42(0.78–2.62)	1.58(0.85–2.95)	27	0.50(0.30–0.80)	0.53(0.32–0.88)	55	0.80(0.54–1.17)	0.92(0.61–1.37)
P-value for trend			0.542	0.361		0.016	0.035		0.128	0.398
Glycitein (mg/day)										
Q1 (<0.85)	468	17	1.00	1.00	47	1.00	1.00	77	1.00	1.00
Q2 (0.85-<1.38)	468	43	2.43(1.36–4.33)	2.66(1.48–4.78)	48	0.98(0.64–1.50)	1.08(0.70–1.67)	100	1.26(0.91–1.74)	1.40(0.99–1.96)
Q3 (1.38-<2.21)	468	30	1.68(0.91–3.09)	1.84(0.99–3.42)	66	1.34(0.90–1.99)	1.46(0.97–2.20)	107	1.33(0.97–1.84)	1.47(1.05–2.06)
Q4 (≥2.21)	468	23	1.30(0.68–2.46)	1.48(0.77–2.85)	17	0.35(0.20–0.61)	0.40(0.22–0.71)	36	0.45(0.30–0.68)	0.53(0.35–0.82)
P-value for trend			0.660	0.989		< .001	0.004		< .001	0.002

^a^ Adjusted by age, education, alcohol consumption, and regular exercise

^b^ Test for trend calculated with the median intake for each category of soybean products or isoflavone intake as a continuous variable

**Table 4:** Odds ratios (OR) and 95% confidence intervals (Cl) of colorectal cancer risk by subsites in relation to intake of soy products and isoflavones in women.

	Control (N = 894)	Proximal colon (N = 53)			Distal colon (N = 113)			Rectum (N = 124)		
	No	No	Age-adjusted OR(95%CI)	Multivariate OR^a^(95%CI)	No	Age-adjusted OR(95%CI)	Multivariate OR^a^(95%CI)	No	Age-adjusted OR(95%CI)	Multivariate OR^a^(95%CI)
Total soy products (g/day)										
Q1 (<42.77)	224	9	1.00	1.00	23	1.00	1.00	37	1.00	1.00
Q2 (42.77-<70.29)	223	19	2.08(0.92–4.71)	1.96(0.85–4.51)	33	1.45(0.82–2.55)	1.39(0.77–2.50)	41	1.11(0.68–1.80)	1.05(0.63–1.76)
Q3 (70.29-<113.66)	223	17	1.86(0.81–4.27)	1.97(0.84–4.61)	39	1.71(0.99–2.97)	1.86(1.04–3.31)	33	0.89(0.54–1.48)	0.96(0.56–1.64)
Q4 (≥113.66)	224	8	0.86(0.33–2.29)	1.01(0.37–2.72)	18	0.79(0.41–1.51)	0.91(0.46–1.78)	13	0.35(0.18–0.67)	0.41(0.20–0.81)
P-value for trend ^b^			0.336	0.646		0.280	0.667		0.001	0.006
Legumes (g/day)										
Q1 (<1.22)	223	21	1.00	1.00	43	1.00	1.00	52	1.00	1.00
Q2 (1.22-<2.40)	224	16	0.75(0.38–1.48)	0.87(0.43–1.76)	24	0.55(0.33–0.95)	0.63(0.36–1.10)	32	0.61(0.38–0.99)	0.70(0.42–1.16)
Q3 (2.40-<5.82)	223	9	0.41(0.19–0.93)	0.47(0.21–1.08)	20	0.46(0.26–0.82)	0.51(0.28–0.92)	21	0.40(0.23–0.69)	0.45(0.25–0.79)
Q4 (≥5.82)	224	7	0.31(0.13–0.76)	0.35(0.14–0.86)	26	0.60(0.35–1.01)	0.63(0.36–1.11)	19	0.36(0.20–0.63)	0.38(2.11–0.70)
P-value for trend ^b^			0.020	0.030		0.345	0.376		0.003	0.007
Tofu (g/day)										
Q1 (<18.73)	224	9	1.00	1.00	15	1.00	1.00	32	1.00	1.00
Q2 (18.73-<32.68)	223	22	2.44(1.10–5.42)	2.71(1.20–6.15)	33	2.21(1.17–4.19)	2.49(1.28–4.83)	37	1.16(0.77–1.93)	1.31(0.76–2.24)
Q3 (32.68-<54.91)	223	9	1.00(0.39–2.56)	1.16(0.44–3.04)	37	2.49(1.33–4.66)	2.81(1.46–5.42)	45	1.41(0.87–2.31)	1.63(0.96–2.75)
Q4 (≥54.91)	224	13	1.42(0.59–3.39)	1.54(0.63–3.77)	28	1.88(0.98–3.61)	2.04(1.03–4.05)	10	0.31(0.15–0.65)	0.34(0.16–0.73)
P-value for trend ^b^			0.831	0.958		0.271	0.220		0.002	0.005
Soy milk (g/day)										
None (= 0)	562	30	1.00	1.00	75	1.00	1.00	81	1.00	1.00
Low (0<-19.1)	166	15	1.69(0.89–3.22)	2.05(1.05–4.00)	25	1.13(0.70–1.83)	1.33(0.80–2.23)	26	1.09(0.68–1.75)	1.29(0.78–2.14)
High (≥19.1)	166	8	0.89(0.40–1.99)	1.02(0.45–2.32)	13	0.59(0.32–1.09)	0.70(0.37–1.33)	17	0.71(0.41–1.23)	0.82(0.46–1.47)
P-value for trend ^b^			0.567	0.756		0.071	0.205		0.190	0.404
Sprouts (g/day)										
Q1 (<5.21)	223	18	1.00	1.00	35	1.00	1.00	36	1.00	1.00
Q2 (5.21-<11.24)	224	14	0.77(0.38–1.60)	0.75(0.36–1.58)	37	1.05(0.64–1.73)	1.02(0.60–1.72)	40	1.10(0.68–1.80)	1.06(0.63–1.77)
Q3 (11.24-<21.86)	223	14	0.77(0.38–1.60)	0.73(0.35–1.55)	30	0.86(0.51–1.45)	0.80(0.46–1.39)	30	0.83(0.49–1.39)	0.77(0.44–1.33)
Q4 (≥21.86)	224	7	0.38(0.16–0.94)	0.39(0.16–0.97)	11	0.31(0.16–0.63)	0.32(0.15–0.66)	18	0.50(0.27–0.90)	0.50(0.27–0.94)
P-value for trend ^b^			0.038	0.047		0.001	0.001		0.007	0.012
Fermented soy paste (g/day)										
Q1 (<2.08)	223	9	1.00	1.00	17	1.00	1.00	26	1.00	1.00
Q2 (2.08-<4.50)	224	15	1.65(0.71–3.85)	1.56(0.66–3.71)	30	1.76(0.95–3.29)	1.71(0.89–3.27)	37	1.42(0.83–2.42)	1.39(0.79–2.44)
Q3 (4.50-<8.70)	223	16	1.75(0.76–4.05)	1.60(0.67–3.78)	36	2.14(1.16–3.92)	2.00(1.06–3.78)	32	1.23(0.71–2.13)	1.16(0.65–2.08)
Q4 (≥8.70)	224	13	1.39(0.58–3.34)	1.24(0.50–3.06)	30	1.79(0.95–3.37)	1.55(0.80–3.00)	29	1.11(0.63–1.96)	0.98(0.54–1.80)
P-value for trend ^b^			0.797	0.986		0.231	0.543		0.859	0.547
Isoflavone (mg/day)										
Q1 (<8.08)	223	9	1.00	1.00	22	1.00	1.00	38	1.00	1.00
Q2 (8.08-<13.83)	224	23	2.52(1.14–5.58)	2.46(1.09–5.55)	35	1.59(0.90–2.80)	1.67(0.92–3.02)	42	1.10(0.68–1.77)	1.11(0.67–1.85)
Q3 (13.83-<22.35)	224	14	1.50(0.63–3.54)	1.57(0.65–3.79)	35	1.60(0.90–2.82)	1.72(0.95–3.14)	25	0.65(0.38–1.11)	0.69(0.39–1.22)
Q4 (≥22.35)	223	7	0.75(0.27–2.05)	0.89(0.32–1.11)	21	0.96(0.51–1.81)	1.16(0.60–2.26)	19	0.49(0.27–0.88)	0.60(0.32–1.11)
P-value for trend ^b^			0.140	0.325		0.568	0.931		0.004	0.035
Daidzein (mg/day)										
Q1 (<3.38)	223	10	1.00	1.00	22	1.00	1.00	39	1.00	1.00
Q2 (3.38-<5.89)	224	21	2.06(0.95–4.48)	2.03(0.91–4.52)	35	1.59(0.90–2.81)	1.71(0.94–3.10)	44	1.12(0.70–1.79)	1.15(0.70–1.91)
Q3 (5.89-<9.89)	224	14	1.35(0.58–3.11)	1.51(0.64–3.56)	33	1.51(0.85–2.68)	1.71(0.93–3.13)	21	0.53(0.30–0.93)	0.59(0.33–1.08)
Q4 (≥9.89)	223	8	0.77(0.30–1.99)	0.87(0.33–2.32)	23	1.06(0.57–1.97)	1.23(0.64–2.35)	20	0.50(0.28–0.90)	0.59(0.32–1.08)
P-value for trend ^b^			0.178	0.346		0.670	0.960		0.003	0.022
Genistein (mg/day)										
Q1 (<3.56)	224	9	1.00	1.00	23	1.00	1.00	34	1.00	1.00
Q2 (3.56-<6.08)	223	20	2.20(0.98–4.95)	2.24(0.98–5.14)	31	1.36(0.77–2.41)	1.45(0.80–2.63)	43	1.27(0.78–2.06)	1.32(0.79–2.22)
Q3 (6.08-<9.79)	224	17	1.84(0.80–4.23)	1.83(0.78–4.29)	34	1.49(0.85–2.63)	1.49(0.82–2.70)	30	0.88(0.52–1.49)	0.87(0.50–1.52)
Q4 (≥9.79)	223	7	0.75(0.27–2.06)	0.90(0.32–2.53)	25	1.11(0.61–2.02)	1.35(0.71–2.54)	17	0.50(0.27–0.92)	0.61(0.32–1.16)
P-value for trend ^b^			0.233	0.466		0.954	0.495		0.006	0.044
Glycitein (mg/day)										
Q1 (<0.93)	224	12	1.00	1.00	28	1.00	1.00	40	1.00	1.00
Q2 (0.93-<1.43)	223	18	1.49(0.70–3.17)	1.68(0.78–3.65)	24	0.86(0.48–1.53)	0.99(0.54–1.80)	35	0.87(0.53–1.42)	1.00(0.59–1.67)
Q3 (1.43-<2.44)	224	16	1.31(0.61–2.84)	1.46(0.66–3.23)	43	1.55(0.93–2.58)	1.71(1.00–2.94)	41	1.02(0.64–1.64)	1.14(0.69–1.88)
Q4 (≥2.44)	223	7	0.57(0.22–1.47)	0.69(0.26–1.83)	18	0.65(0.35–1.20)	0.77(0.40–1.48)	8	0.20(0.09–0.43)	0.24(0.11–0.54)
P-value for trend ^b^			0.253	0.420		0.146	0.433		< .001	0.001

^a^ Adjusted by age, education, alcohol consumption, and regular exercise

^b^ Test for trend calculated with the median intake for each category of soybean products or isoflavone intake as a continuous variable

In the analyses by the menopausal status of women, a decreased risk for colorectal cancer among the highest quartile intake groups for total soy products, legumes, sprouts, and glycitein was observed only among postmenopausal women (Table 5).

**Table 5:** Odds ratios (OR) and 95% confidence intervals (Cl) for colorectal cancer risk in relation to intake of soy products and isoflavones by menopausal status.

	Premenopausal women (n = 346)			Postmenopausal women (n = 846)		
	Controls /cases(n)	Age-adjusted OR(95%CI)	Multivariate OR^a^(95%CI)	Controls/cases(n)	Age-adjusted OR(95%CI)	Multivariate OR(95%CI)
Total soy products (g/day)						
Q1 (<42.77)	85/24	1.00	1.00	139/48	1.00	1.00
Q2 (42.77-<70.29)	71/22	1.14(0.59–2.20)	1.02(0.51–2.05)	152/74	1.41(0.92–2.18)	1.45(0.89–2.36)
Q3 (70.29-<113.66)	66/21	1.17(0.60–2.29)	1.12(0.55–2.27)	157/70	1.30(0.84–2.01)	1.53(0.94–2.51)
Q4 (≥113.66)	42/15	1.31(0.62–2.77)	1.06(0.47–2.37)	182/24	0.38(0.22–0.66)	0.52(0.29–0.95)
P-value for trend ^b^		0.479	0.853		< .001	0.009
Legumes (g/day)						
Q1 (<1.22)	86/42	1.00	1.00	137/78	1.00	1.00
Q2 (1.22-<2.40)	69/18	0.54(0.29–1.03)	0.66(0.34–1.30)	155/56	0.63(0.42–0.96)	0.68(0.43–1.10)
Q3 (2.40-<5.82)	60/13	0.45(0.22–0.90)	0.52(0.25–1.08)	163/37	0.40(0.25–0.63)	0.42(0.25–0.70)
Q4 (≥5.82)	49/9	0.39(0.17–0.87)	0.43(0.18–1.03)	175/45	0.45(0.29–0.69)	0.46(0.28–0.75)
P-value for trend ^b^		0.047	0.080		0.007	0.015
Tofu (g/day)						
Q1 (<18.73)	71/18	1.00	1.00	153/41	1.00	1.00
Q2 (18.73-<32.68)	79/25	1.26(0.63–2.51)	1.41(0.68–2.90)	144/70	1.82(1.16–2.84)	2.06(1.24–3.42)
Q3 (32.68-<54.91)	61/23	1.46(0.72–2.96)	1.57(0.74–3.31)	162/69	1.59(1.02–2.49)	1.94(1.17–3.24)
Q4 (≥54.91)	53/16	1.25(0.58–2.68)	1.07(0.47–2.42)	171/36	0.79(0.48–1.30)	0.96(0.55–1.67)
P-value for trend ^b^		0.621	0.996		0.044	0.245
Soy milk (g/day)						
None (= 0)	178/52	1.00	1.00	384/137	1.00	1.00
Low (0<-19.1)	46/19	1.43(0.77–2.65)	1.44(0.74–2.81)	120/51	1.19(0.81–1.74)	1.52(0.98–2.35)
High (≥19.1)	40/11	0.94(0.45–1.96)	0.93(0.42–2.04)	126/28	0.62(0.40–0.98)	0.76(0.46–1.26)
P-value for trend ^b^		0.740	0.724		0.025	0.160
Sprouts (g/day)						
Q1 (<5.21)	69/21	1.00	1.00	154/71	1.00	1.00
Q2 (5.21-<11.24)	71/26	1.16(0.60–2.27)	1.29(0.63–2.63)	153/68	0.96(0.65–1.44)	0.89(0.56–1.40)
Q3 (11.24-<21.86)	69/20	0.96(0.48–1.95)	0.97(0.46–2.03)	154/56	0.79(0.52–1.20)	0.70(0.43–1.13)
Q4 (≥21.86)	55/15	0.89(0.42–1.89)	0.92(0.42–2.03)	169/21	0.27(0.16–0.46)	0.26(0.14–0.47)
P-value for trend ^b^		0.604	0.607		< .001	< .001
Fermented soy paste (g/day)						
Q1 (<2.08)	73/25	1.00	1.00	150/27	1.00	1.00
Q2 (2.08-<4.50)	78/25	0.99(0.52–1.86)	0.85(0.43–1.68)	146/61	2.33(1.40–3.87)	2.62(1.49–4.63)
Q3 (4.50-<8.70)	76/17	0.63(0.31–1.26)	0.50(0.23–1.05)	147/69	2.63(1.59–4.34)	2.99(1.70–5.25)
Q4 (≥8.70)	37/13	1.05(0.48–2.29)	0.76(0.32–1.81)	187/59	1.77(1.07–2.94)	1.80(1.02–3.16)
P-value for trend ^b^		0.901	0.415		0.577	0.793
Isoflavone (mg/day)						
Q1 (<8.08)	84/24	1.00	1.00	139/48	1.00	1.00
Q2 (8.08-<13.83)	78/28	1.29(0.69–2.41)	1.27(0.65–2.47)	146/75	1.49(0.97–2.29)	1.57(0.96–2.56)
Q3 (13.83-<22.35)	63/17	1.02(0.51–2.08)	1.04(0.49–2.18)	161/59	1.06(0.68–1.66)	1.24(0.75–2.06)
Q4 (≥22.35)	39/13	1.19(0.51–2.08)	1.04(0.45–2.41)	184/34	0.54(0.33–0.88)	0.72(0.41–1.25)
P-value for trend ^b^		0.829	0.940		0.001	0.056
Daidzein (mg/day)						
Q1 (<3.38)	90/25	1.00	1.00	133/49	1.00	1.00
Q2 (3.38-<5.89)	69/30	1.59(0.86–2.94)	1.79(0.92–3.48)	155/74	1.30(0.85–2.00)	1.31(0.80–2.13)
Q3 (5.89-<9.89)	63/14	0.87(0.42–1.80)	0.97(0.45–2.08)	161/54	0.91(0.58–1.43)	1.07(0.64–1.79)
Q4 (≥9.89)	42/13	1.13(0.53–2.43)	1.03(0.45–2.37)	181/39	0.59(0.36–0.94)	0.73(0.43–1.26)
P-value for trend ^b^		0.868	0.689		0.002	0.079
Genistein (mg/day)						
Q1 (<3.56)	85/24	1.00	1.00	139/45	1.00	1.00
Q2 (3.56-<6.08)	73/28	1.41(0.75–2.65)	1.43(0.73–2.78)	150/69	1.42(0.92–2.21)	1.54(0.93–2.54)
Q3 (6.08-<9.79)	64/18	1.05(0.52–2.10)	1.02(0.49–2.11)	160/65	1.26(0.81–1.97)	1.37(0.83–2.28)
Q4 (≥9.79)	42/12	1.08(0.49–2.37)	0.93(0.40–2.17)	181/37	0.63(0.39–1.03)	0.86(0.50–1.49)
P-value for trend ^b^		0.956	0.690		0.009	0.244
Glycitein (mg/day)						
Q1 (<0.93)	73/23	1.00	1.00	151/61	1.00	1.00
Q2 (0.93-<1.43)	81/20	0.79(0.40–1.55)	0.74(0.36–1.51)	142/58	1.01(0.66–1.55)	1.30(0.80–2.12)
Q3 (1.43-<2.44)	64/24	1.24(0.64–2.41)	1.21(0.60–2.45)	159/79	1.23(0.82–1.84)	1.48(0.94–2.35)
Q4 (≥2.44)	46/15	1.09(0.51–2.31)	1.02(0.45–2.28)	178/18	0.25(0.14–0.44)	0.32(0.17–0.60)
P-value for trend ^b^		0.795	0.898		< .001	0.001

^a^ Adjusted by age, education, alcohol consumption, and regular exercise

^b^ Test for trend calculated with the median intake for each category of soybean products or isoflavone intake as a continuous variable.

## Discussion

Although the middle quartiles of intake was associated with an elevated risk, the highest intake quartile of total soy products or dietary isoflavones is associated with a reduced risk for overall colorectal cancer, and the association might be more relevant to distal colon or rectal cancers. The reduced risks for colorectal cancer among high intake groups were most consistent for legumes and sprouts in both men and women and for all subsites.

A meta-analysis of epidemiological studies on soy food intake and colorectal cancer risk showed a 21% reduction in the colorectal cancer risk among the high intake groups in women (the combined risk estimate: 0.79, 95% CI: 0.65–0.97), whereas there was no apparent benefit from soy consumption on the colorectal cancer risk in men (the combined risk estimate: 1.10, 95% CI 0.90–1.33).[13] The subgroup analysis for the types of soy products or study population did not show significant heterogeneity in the pooled risk estimates.[13] Among soy foods, tofu[23–28] and miso soup[14, 25, 28–30] have been the most widely analyzed. We found a consistent association between a high intake of legumes and a reduced risk for overall colorectal cancer, and a high intake of fermented soy paste was associated with an increased risk for overall colorectal cancer as well as cancer of all the subsites in men. Legumes contain carbohydrates (6–62%), proteins (20–30%), and fiber (3–31%), depending on the type.[31] Legumes are important sources of minerals such as iron, zinc, calcium, and selenium as well as nonnutrient compounds such as lectins, agglutinins, protease inhibitors, bioactive peptides, phenolic compounds (tannins), and saponins.[31] Additionally, soybeans are an important source of linoleic and linolenic acids.[31] Fermented soy paste contains high salt, however, and there is some limited epidemiological evidence that a high salt intake has a role in colorectal carcinogenesis.[32]

Genistein, the most predominant isoflavone, is known to inhibit prostate carcinogenesis in mouse models, to modulate genes that are involved in the control of the cell cycle and apoptosis, and to have antioxidant properties.[33] In addition, equol, a plant isoflavone metabolite produced by ruminal bacteria of mammalian, has a weak estrogenic effect, which has been linked to a protective effect against breast carcinogenesis. [34]Among nine epidemiological studies on isoflavone intake and the colorectal cancer risk, four case-control studies and one prospective cohort study reported a reduced risk for colorectal cancer among high intake groups,[15, 16, 18, 35, 36] whereas three cohort studies did not find any association.[14, 17, 37] A Japanese case-control study suggested a beneficial effect of isoflavones on the colorectal cancer risk only among postmenopausal women.[38]

Two studies on gender–specific association, decreased risk of colorectal cancer was observed only among women for soy food. [17, 39] In a case-control study conducted in Hong Kong, women who consumed soy products 4 times per week or more showed decreased risk for colorectal cancer (odds ratio (95% CI): 0.47 (0.28–0.81)), whereas statistical significance was not reached among men (odds ratio (95% CI): 0.66 (0.40–1.08). [39] In a Japanese cohort study, high intakes of total soy food (hazard ratio (95% CI): 1.24 (0.77–2.00) for men and 0.56 (0.34–0.92) for women: the highest tertile vs. the lowest) or isoflavones (hazard ratio (95% CI): 1.47 (0.90–2.40) for men and 0.73 (0.44–1.18) for women: the highest tertile vs. the lowest) was associated with decreased risk of colon cancer only among women. [17] Other studies, however, did not find a significant difference in risk between men and women.[14, 27, 37, 38]A cohort study of Chinese women reported a high intake of soyfoods and a reduced risk of colorectal cancer only among postmenopausal women and in rectal cancer.[18] With the result from a Japanese case-control study,[38] a more prominent association between soy or isoflavone intake and colorectal cancer among postmenopausal women is consistent with the results of this study. The putative biological mechanism that connects postmenopausal colorectal cancer and soy foods is the estrogenic effect of soyfoods.[18] A diphenolic structure of isoflavones is similar to the structure of 17β-estradiol, and various isoflavone compounds bind to estrogen receptor α and ß. [40] An in vitro study showed that isoflavones act as estrogen antagonists with a premenopausal (high) dose of estradiol, whereas they act as estrogen agonist in a low-estrogen environment near the serum level of postmenopausal women.[40]

Few studies have assessed the association between soyfoods or isoflavones and the colorectal cancer risk by subsites.[14, 15] The Fukuoka Colorectal Cancer Study reported an inverse association of soyfoods and isoflavones with rectal cancer in men and overall colorectal cancer in postmenopausal women.[15] The subsites of colorectal cancer show different molecular profiles, such as CpG island methylator phenotypes and microsatellite instability.[41] In addition, the estrogen receptor ß expression in normal epithelium was higher in the ascending colon than in the descending colon,[42] and it might partly explain the right-side dominance in colon cancer in women and the difference in susceptibility to isoflavone intake.[1]

There is substantial variation in the intake level of soyfood and isoflavones among different populations.[43] The median intake levels of isoflavones among the control groups were 12.47 mg/day for men and 13.46 mg/day for women. These intake levels are much higher than those observed in studies from Canada,[16] England,[37] and Italy[36] and comparable or lower than those in studies from Japan[14, 15, 17, 38] and China.[18]

The strengths of this study include detailed information on the main exposures such as different types of soyfoods and isoflavones as well as on the anatomical subsites of colorectal cancer. The limitations include the use of only five soyfood items to calculate the dietary isoflavone intake. Limited numbers of premenopausal women included in our study might lead to low statistical power to detect meaningful association.

The highest intake quartiles of soyfoods or isoflavones are associated with a reduced risk for overall colorectal cancer. In the subsite analysis, a reduced risk among the highest intake groups for soyfoods or isoflavones is more prominent for distal colon cancer in men and rectal cancer in women. Additionally, the protective effects of soyfoods are observed only in postmenopausal women. Although precaution is required for the probably elevated risk among middle intake quartiles, our results suggest a potential role of soyfoods in colorectal cancer prevention, and the effect might differ by colorectal cancer subsite, gender, and menopausal status of women.
